# The market for human smuggling: Structure, mechanisms, and paradoxes

**DOI:** 10.1073/pnas.2511019122

**Published:** 2026-08-10

**Authors:** Paolo Campana

**Affiliations:** ^a^ Institute of Criminology, University of Cambridge, Cambridge CB3 9DA, United Kingdom

**Keywords:** illegal markets, supply chains, coordination, technology, policy dilemmas

## Abstract

Human smuggling is the facilitation of unlawful cross-border movement. It is primarily a commercial activity, offered as a service to willing, paying customers, and a large-scale market catering to millions seeking to circumvent mobility restrictions. Contrary to prevailing media and political narratives that depict smuggling as dominated by powerful and highly sophisticated criminal organizations, this study documents a fragmented, decentralized market composed of independent, localized actors operating through flexible, often temporary networks. These flexible arrangements, coupled with low barriers to entry, confer resilience and adaptability to the market, making enforcement difficult and resource-intensive. Intensified enforcement often produces unintended consequences, including heightened migrant vulnerability and increased reliance on smugglers. Despite regional differences, smuggling operations exhibit striking structural similarities shaped by local knowledge and monitoring costs. Such enduring structural characteristics exist alongside a growing role played by communication technologies, which are reshaping dynamics between service providers and customers. Ultimately, human smuggling remains a market fraught with paradoxes: Migrants face severe risks yet view smugglers as essential facilitators, while states confront the complex task of balancing border enforcement with humanitarian obligations and protections of vulnerable individuals.

Analytically speaking, human smuggling is the facilitation of unlawful cross-border movement for material gain. The service provided is an illegal border crossing (IBC). More broadly, we can understand human smuggling as a commercial market for IBCs in which two main sets of actors operate: those who offer IBCs, collectively defined as “smugglers” (supply side), and those who wish to purchase IBCs, often defined as “migrants” (demand side).^*^ Motivations for enlisting the services of smugglers can vary and depend on the individual circumstances of each migrant. These often include leaving war zones, avoiding persecution, escaping poverty and dwindling natural resources, or looking for a better life.

The terms human smuggling and human trafficking are often confused, but they represent two very different phenomena, albeit potentially occurring in the same places and populations. While a victim of trafficking and a genuine client of a smuggler might find themselves shoulder-to-shoulder in the same inflatable boat, thus sharing the same means to cross the same border, smuggling and trafficking remain distinct—both analytically and legally. In the case of smuggling, the service provided is the illegal entry into a country—and a migrant enters into the transaction with a smuggler voluntarily (1). Some may have few options other than to migrate, and in that sense be forced to migrate, but they are not forced into that action by an individual or organization selling them the smuggling service. The relationship between a migrant and a smuggler terminates when the former has reached the agreed destination and paid the latter the agreed sum. On the other hand, the purpose of trafficking is obtaining control over a person for exploitation, including sexual exploitation, labor exploitation, or domestic servitude (2).^†^ Victims of human trafficking may enter a country through legal channels, and in some cases, trafficking takes place wholly within national borders.

The market for human smuggling can be imperfect, risky, overpriced, and subject to constraints stemming from the condition of illegality. However, human smuggling remains fundamentally a form of (illegal) trade in services. The present paper explores the organizational structure and functioning of this market.

## 1. Origins and Motivations of Prohibiting Some Border Crossings

Mobility restrictions are not a new phenomenon, and neither are the operations of smugglers. Individual countries have long had domestic legislation imposing penalties of varying severity on violations of immigration laws. As one among many examples, when the United States enacted the Chinese Exclusion Act in 1882 to restrict Chinese immigration into the country, smugglers started helping people circumvent those restrictions in search of the American dream—in exchange for a fee (3). However, it was only in 2000 that a broad international consensus emerged around the criminalization of human smuggling. Such consensus is crystallized in the Protocol against the Smuggling of Migrants by Land, Sea and Air (1), adopted by the General Assembly of the United Nations in November 2000 as an additional protocol to the Convention Against Transnational Organized Crime signed in Palermo, Italy, in the same year. The Protocol, initially signed by 112 countries and now ratified by a total of 154 parties (as of October 2025), stipulates that States shall adopt legislative measures to establish the smuggling of migrants as a criminal offense. The Protocol also provides a widely agreed definition of migrant smuggling, also referred to in the literature as human smuggling, as “the procurement, in order to obtain, directly or indirectly, a financial or other material benefit, of the illegal entry of a person into a State Party of which the person is not a national or a permanent resident” (Article 3).^‡^

The primary legal justification for illegality is to protect the sovereignty of States and their right to set conditions around the crossing of their borders/presence on their territory, which makes human smuggling primarily an offense against the State (1: Preamble). The same Protocol explicitly utilizes the word “harm” only once, and that is in relation to the harm brought “to the States concerned”. It subsequently also expresses a “concern” that “the smuggling of migrants can endanger the lives or security of the migrants involved” (1: Preamble). There are, therefore, two distinct types of harm connected to human smuggling: to a State and, by extension, its citizens; and to the migrants, who face risks during their journey and are vulnerable to exploitation, violence, and fraud. Migrant flows are mostly from poorer and/or war-torn countries into wealthier and more stable areas, and there can be fears (justified or not) on the part of the citizens of receiving countries that unfettered or unregulated flows could threaten their affluence, social order, or cohesion. This is against the backdrop of flows that are potentially larger than in the past due to the combination of general population growth, growing general affluence and falling costs of transportation, as well as improvements in communication between people and family members in other countries.

## 2. Scale of the Market

**Fig. 1.**
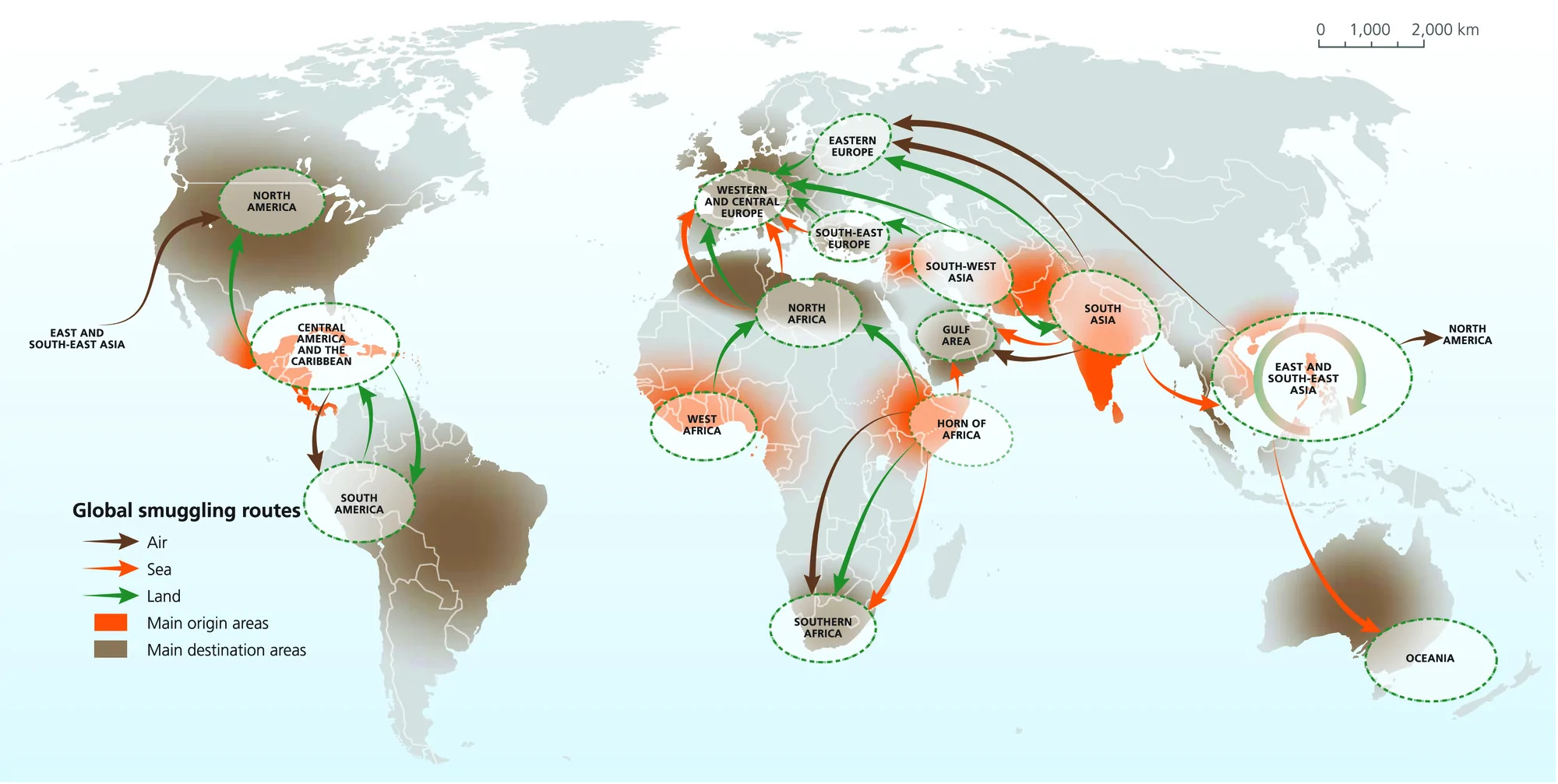
Global smuggling routes. Main land, sea, and air macroroutes of migrant smuggling worldwide, as identified by the UNODC. Each directed arrow indicates a route linking main origin and destination areas. Source: UNODC (4, p. 11). Image credit: Reprinted from ref. 4.

The demand for smuggling services can arise anywhere there is a border with some restrictions to travel (at least for some nationalities), some level of enforcement, and people willing to cross such border who do not possess the requirements for legal entry. Global estimates about the size of the market are rarely produced. The latest available estimate compiled by the United Nations Office on Drugs and Crime (UNODC) is for 2016, and puts the figure at a minimum of 2.5 million individuals smuggled (4, pp. 23–24). This is likely very conservative as only a minority of countries worldwide produce statistics on human smuggling (5). Additionally, estimates are produced based on crossings intercepted by the authorities, and the rate of interception varies greatly depending on the level of enforcement but also geographical specificities (e.g., land vs. sea routes). The same report identifies fifteen macroroutes worldwide (Fig. 1); while some subroutes (or local segments) have emerged or disappeared over the last 10 y, the macropicture has remained broadly unchanged.

Of the 15 macroroutes, it is commonly believed that the largest in size (and revenues) is the route into North America via the southern US border, accounting for roughly one in three migrants smuggled globally in 2016. Crossings into Europe via its southern Mediterranean borders accounted for roughly 15% of the global smuggling in the same year. If movements from East Asia into Europe as well as from West and East Africa to North African countries are included—on the assumption that they may have Europe as final destination—then Europe is associated with roughly 40% of the global smuggling. However, such figures might also reflect data availability, with crossings into the United States and Europe being the better documented.

It is also possible that, since 2016, the global market for migrant smuggling has increased in size. For instance, just for the Mexico–US border, the number of migrants apprehended by the authorities in 2024 was double the number estimated by UNODC in 2016.^§^ In the four post-Covid fiscal years (2021–2024), some 4.8 million IBCs were detected by the US authorities at the Southern border with Mexico; over the 10 y 2015–2024, this number totaled 7.4 million.^¶^ In the same post-Covid period (2021–2024), European authorities detected 784,157 IBCs on Mediterranean routes into Italy, Greece, and Spain, and some 2.75 millions over the 10 y 2015–2024.^#^

Estimating revenues associated with human smuggling is even more speculative. UNODC puts the *minimum* annual global revenue at between US$5.5-7 billion in 2016, with the US route generating between US$3.7-4.2 billion and all three Mediterranean routes between US$320 and 500 million (4, pp. 22–23). Taking a conservative approach, we can calculate an updated estimate of overall revenues for the US route based on US$5,000 as the average fee, an apprehension rate of 75% (the Department of Homeland Security [DHS] estimates that apprehensions might be in the region of 70 to 80%) and that family units pay a single fee (see ref. 6, p. 3 for data on family units). This gives us a ballpark figure in the region of US$8 billion; this goes up to US$12.8 billion if we take the higher-end estimate of US$8,000 as the average fee.^||^ A more recent UNODC estimate (2023) for the Central Mediterranean route alone upped the 2016 figure to between US$290-370 million (7, p. 2). French authorities estimate that small boat crossings between France and the United Kingdom have generated revenues of about €150 million in 2022 (US$175 million).^**^ In Africa, the Mixed Migration Centre (8, p. 6) estimates that smuggling people from East and West Africa to Libya and Tunisia has generated US$60.5 million in revenues in 2021.

As for the use of smugglers to assist unlawful crossings, multiple sources converge in showing that “smugglers usage” is very high for routes into wealthy areas of the world, reflecting a correspondingly higher level of border enforcement by such countries. For example, between 90% and 95% of migrants smuggled into the United States have relied on a smuggler according to both law enforcement data (9) and academic surveys^††^; this was the case for 92% of migrants identified by the European Border and Coast Guard Agency (Frontex) at the Greek, Italian, and Spanish borders (10), 90% of migrants interviewed in Europe by Mixed Migration Centre (4Mi data: 11) and 100% of migrants smuggled into the Middle East (4Mi data: 11)^‡‡^. In other parts of the world, smuggling usage is less prevalent, but still far from negligible: It is estimated at 78% in Asia; at between 67 to 49% in Africa (higher in Northern African routes and lower in Southern African routes) and at 41% in Latin America—the lowest smuggler usage rate (4Mi data: 11).

## 3. Structure and Nature of the Illegal Market, Supply Chain, and Criminal Enterprises

The tragic death of 39 Vietnamese migrants in a refrigerated trailer transported by ferry from Belgium to the United Kingdom in October 2019 sadly provides detailed evidence about the organization of (long-distance) smuggling. During the trial, Witness X–who had completed the same journey organized by the same individuals 12 d before the tragedy—testified that he left Vietnam by plane in February 2019. His flight to Poland was made possible by a visa issued following enrollment on a fictitious business course—both organized by a first smuggler for some £20,000 (US$24,500; 12, 13)^§§^. Once in Poland, Witness X contacted a separate London-based Vietnamese smuggler named Phong via the Viber messaging app. Pong agreed to organize the onward journey to the United Kingdom for £13,000 (US$16,000). There is no evidence of a connection between the London-based smuggler and the first smuggler. Following Pong’s instructions, Witness X traveled to an apartment on the outskirts of Paris where 13 other Vietnamese individuals were gathered. They were brought by cars to a rendezvous point in Northern France, loaded into a trailer, and then driven to Zeebrugge in Belgium. After crossing the Channel, the trailer was collected by another driver in Essex. Not far from the port, the migrants finally got into a fleet of waiting black Mercedes cars and were brought to the flat of Phong in South London, where Witness X was held until his parents paid the fee for the cross-Channel journey (12, 13).

Evidence presented in court in the United Kingdom, France, and Belgium sheds further light on the smugglers’ modus operandi. It points to the presence of small clusters of individuals specialized in a specific task, e.g., organizing sea crossings using trailers (this task was carried out mainly by English, Irish, and Romanian nationals working in the hauling industry)^¶¶^, managing safe houses (mostly Vietnamese nationals)^##^ or providing trips to the rendezvous points with the truck drivers (primarily French, Algerian, and Moroccan nationals working as taxi drivers)^##^. A similar system of safe houses run by Vietnamese nationals was also uncovered in Belgium and Germany (14, pp. 6 and 28). Haulers, taxi drivers, and safe house runners are likely to be all independent actors contracted as service providers on a repeated basis—loosely connected through horizontal, transactional interactions (Crossing the Channel in sealed containers is marketed at a much higher price as the chances of detection are considered lower than open lorries and small boats. A key element in the October 2019 fatal journey is that two lorries were expected to pick up the 41 migrants in the small town of Bierne in Northern France (two people arrived late at the meeting point, thus missing the onward leg). However, only one lorry showed up at the small farm in the countryside. To not lose the money and fulfil the terms of their “contract,” the individuals tasked with organizing this stage of the journey made the fatal decision to place all 39 migrants in one single sealed container (details about the operations are reported in ref. 15)).

Smugglers often meet the legal definition of being an organized crime group—because multiple individuals are often cooperating over a period of time—but it is common to overstate the size and market power of smuggling organizations. The evidence reviewed in this section suggests that it would be quite unusual for one organization to literally monopolize all smuggling service delivery on a particular route, or even for a few large firms to enjoy an oligopoly. Instead, it appears more common that multiple smuggling service providers operate in parallel and hence in competition along the same route.

For instance, no evidence of market monopolization has been found on the US–Mexico border (16, 17). Smuggling operations are predominantly small-scale and localized, involving “thousands” of smugglers who either work independently or form “loose partnerships” with other smugglers and groups (18, p. 152; also refs. 16 and 19). There is evidence of division of labor, with smugglers, or small groups of smugglers, performing specialized tasks, e.g., acting as recruiters, lookouts, drivers, or guides (16, 18–20). Long-distance smuggling between China and the United States in the 1990s showed a markedly similar organizational structure—characterized by independent groups with no one exerting dominance or monopoly (21). Smuggling was carried out by decentralized, horizontal associations of a few closely connected individuals (22).

Routes into Europe display similar organizational patterns. Smuggling from West and East Africa into Southern Europe via North African countries (Central Mediterranean route) is conducted by largely independent (groups of) smugglers, collaborating across stages along the journey while often competing within the same stage (23–25). Available evidence indicates neither monopolization along these routes nor that single groups provide IBCs throughout the entire journeys. On the contrary, the market is characterized by several small—very rudimentary—localized hierarchies built around more entrepreneurial and resourceful smugglers operating at different stages along the route (23; see also ref. 25). Long-distance routes into Europe via Türkiye show a similar structure, with hundreds of small units operating independently and loosely linked in flexible, horizontal networks (26–30). In short, studies on both the Central and Eastern Mediterranean routes found evidence of groups with limited (if any) internal hierarchy, a clear local dimension, and operating through flexible partnerships, often lasting for relatively short periods (23, 29, 30).

In sum, evidence across different routes *and* periods converges in suggesting that a) for any route or route segment, there are multiple service providers and not a monopolist, and b) there tends to be little vertical integration (if any) across different segments along a route. The market for human smuggling is highly fragmented and populated by a great number of organizations that are typically small in size and rudimentary in hierarchy.

### 3.1. Monitoring Costs and Local Knowledge.

Why do smugglers operating along routes that are so different in legal, physical, and cultural environments tend to adopt similar organizational structures? One theory is that this is the consequence of two factors: a) the costs of monitoring employees grow with organization size and b) the importance of local knowledge reduces opportunities for efficiency gains from one centralized entity operating in multiple local markets (Low capital requirements further diminish the need for substantial financing that could typically be sustained only by large entities).

Smuggling organizations need to monitor the performance (and honesty) of their members, which in turn generates costs. The farther away these members operate, the greater the monitoring costs. Similarly, smugglers need to track the whereabouts of their customers in order to deliver the service and, crucially, to collect the fee. The broader the geographic scope of an organization, the higher the monitoring costs. This is a common problem for all actors operating under conditions of illegality, as argued by Reuter (31, p. 21). Reuter further maintained that market illegality creates strong incentives for segmentation (31, p. 9). Human smuggling is no exception. By segmenting the market geographically and operationally, smugglers can keep both types of monitoring costs low. Segmentation also allows smuggling organizations to handle a much larger number of customers, making it more profitable to turn around high numbers of customers over a shorter distance/single border when demand is robust (Contrary to human trafficking organizations, which are largely limited in their scale by monitoring costs (32)).

Local knowledge is a key factor of production in human smuggling. Different types of knowledge play a role: a) about the physical characteristics of a border (e.g., walking trails, safe rest areas); b) about the strategies adopted by law enforcement (types and timing of patrols, etc.); c) about the presence and accessibility of corrupt officials; d) about the availability of resources to support the crossing (e.g., warehouses to store equipment and safe houses to accommodate migrants in transit). All these different types of knowledge are localized and border-specific. The cost of acquiring such knowledge increases with the number of borders crossed (i.e., as an organization’s reach expands), and the potential for achieving significant economies of scale by adding borders remains limited. As a result, the localized nature of knowledge incentivizes smugglers to specialize in a specific border (Not all borders are equal: some border crossings are easier to facilitate as they only require acquisition of knowledge; for others, smugglers need to provide some (rudimental) infrastructure for the crossing. Arguably, one of the largest investment that smugglers need to face is the acquisition of boats for sea crossings. In the case of small boat crossings between France and the UK, for example, the cost of purchasing an inflatable boat and the logistics involved in storing it far away from the highly policed beaches and bringing it to the sea *just in time* for the crossing is estimated at €10,000, which is around 10% of the revenues generated by each crossing (33, p. 10)).

Technology can be employed to guide and monitor migrants, as well as to foster coordination among (and control of) smugglers from afar. However, while technology is indeed used to assist crossings and foster coordination, it does not appear to reduce monitoring costs enough to significantly alter the incentives for market segmentation. Additionally, while technology enables cheaper collection and sharing of information, it appears unable to generate multiborder economies of scale. In short, technology appears to have facilitated horizontal coordination more than it has fostered vertical integration.

### 3.2. Illegal Governance and Human Smuggling.

Human smugglers do not operate in a vacuum but alongside other illegal actors. An important set of such actors are Mafia-type organizations, militias and armed groups, as well as the so-called “cartels” in the case of the Mexican border. What characterizes such actors is that they are involved in the illegal governance of markets and territories (31, 34–36). The relationship between smugglers and governance actors is akin to the one merchants have with a State: They pay for the right to trade on the latter’s territory.

Mafias, armed groups, militias, Mexican cartels as well as corrupt officers are (illegal) governance actors; smugglers are merchants. In Libya, for instance, armed groups provided protection against competition from rival smugglers for a fee (37), while in Mali, armed groups levy fees on passage and provide paid escorts (38). In Colombia, the Gaitanista Self-Defence Forces (AGC), a paramilitary group, charges a 10% tax on both formal and informal businesses transporting migrants across the Gulf of Urabá and the guides who accompany them through the Darién Gap to the border with Panama, providing some degree of protection in exchange (39). Corrupt law enforcement officers can also provide illegal governance and protection to smugglers, as in the case of Niger (40). In the case of Mexico, the so-called cartels (more formally labeled as Drug Trafficking Organizations, or DTOs) often charge a tax, called *el piso* or *derecho de piso*, to smugglers operating in the territory they control. This is a “tax” paid for the right to pass through a territory (16, 41–43). According to Izcara Palacio, Mexican cartels have in recent years increased the tax imposed on human smugglers to make up for diminishing returns in their drug business, significantly driving up smugglers’ operating costs and straining their finances (16). At the same time, heightened border surveillance has further driven up such costs, as smugglers must now spend more on bribes (16). In the Altar Valley, Mexico, criminal groups controlling territories close to the border are seeking to extract more resources from smugglers, leading to violent clashes between groups controlling different parts of the valley (44).

To what extent are illegal governance actors also directly involved in smuggling? In Europe, there is no evidence that the Sicilian Mafia is involved in smuggling migrants into Sicily and then onward toward Northern Italy and beyond (23). Similarly, İçduygu found no indication of Mafia-type organizations participating in migrant smuggling in Türkiye (45). Zhang and Chin observed no generalized direct involvement of traditional Chinese organized crime groups in human smuggling into the United States (22). Several authors maintained that Mexican DTOs have typically not been *directly* involved in human smuggling activities (16, 41). Following increased enforcement and diminishing returns of drug trafficking activities, human smuggling is emerging as an economic alternative for local actors previously involved in drug trafficking as, for example, in the Altar Valley, Mexico, pointing to a switch between the type of transborder trade (44) (A switch between commodities has also been documented in Zuwara, Libya. Following a crackdown on smuggling activities by the local council, some migrant smugglers switched to smuggling fuel or subsidised food items (37, pp. 24 and 68)). More fieldwork research is needed to fully understand to what extent diversification strategies among Mexican DTOs and other actors are emerging trends or isolated cases.

## 4. How Smugglers and Migrants Navigate Illegality: Mechanisms of Market Exchange

Underpinning a fragmented market composed of thousands of largely independent smugglers and hundreds of thousands of migrants, there are literally millions of small transactions between customers and service providers. They are successfully completed despite challenging conditions generated by market illegality: the absence of formal mechanisms to enforce contracts, ensure compliance, and solve disputes (31, 35); the imperfect quality and limited availability of information, and the related difficulties and risks in advertising products and reputations (35, 46); and the difficulties in creating a credit market as well as recover debts (35). The greater the intensity of enforcement, the greater the need to rely on a smuggler (47, p. 1590); at the same time, higher enforcement exacerbates challenges. The market for smuggling services is characterized by an acute information asymmetry problem, similar to the market for lemons (used cars) studied by Akerlof (48): Smugglers hold much more information about the quality (and safety) of their services than do their potential clients (migrants). This is exacerbated by the absence of any formal, third-party entity tasked with checking the quality of the service provided, the veracity of the information conveyed by smugglers, or the latter’s credentials—as a consequence, once again, of market illegality. Yet, transactions do take place. What strategies have smugglers and migrants developed to overcome such challenges?

We frequently observe suppliers of human smuggling services attending to their reputation. This is consistent with the fact that human smuggling services are what economists term an experience good: one whose quality is unobservable before purchase and is not purchased repeatedly (At the very least, not from the same smuggler). In addition, the market for human smuggling is competitive and characterized by the presence of multiple smugglers offering similar services at comparable prices (49). Smugglers invest time and resources in building a reputation for reliability and competence (21, 50, 51). There are documented cases of top-level Chinese smugglers contributing money to their hometown, including to improve local infrastructure (21). In one case, a smuggler operating along the route from the Horn of Africa to Italy was wiretapped stating that he had paid compensation to the families of those who died in a shipwreck in the Mediterranean (23). Reputations are circulated not only through traditional word-of-mouth (50), but increasingly also via social media, where smugglers promote themselves through carefully crafted advertisements and migrants exchange information (4, 52) (Physical hubs such as specific neighbourhoods in cities along a route or migrants camps also serve as spaces where services are advertised, information exchanged, reputations built and checked (53, 54)).

Moreover, smugglers may offer multiple-attempt guarantees as a way of signaling competence, reliability, and trustworthiness. In such cases, if a journey is unsuccessful due to, e.g., law enforcement, clients can make further attempts at no additional cost (19, 20, 28, 53, 55). Offering warranties is a strategy that reliable sellers might use to differentiate themselves from less reputable ones, as suggested by Akerlof (48) in his work on second-hand car sellers.

The importance of reputation in the smuggling market aligns with Reuter’s argument that reputation is a vital asset in illegal markets (31, 35). Reuter also argued that reputations in such markets do not travel easily (31). This is reflected and exacerbated in human smuggling, i.e., a market in which customers are unlikely to make repeated transactions with the same smugglers. It is therefore not surprising that smugglers and migrants often come from nearby places or share a similar ethnic background or language (19, 22, 23, 30, 56–58). In this way, migrants can collect and verify information, including on a smuggler’s reputation, more efficiently—and thus make a safer choice of smuggler—while smugglers can leverage community ties with a migrant’s family in case of missing payments. Using survey data from deported Mexican migrants, Hernández Campos and Torre Cantalapiedra show that migrants who contact smugglers on the way to or in a border city face a higher risk of abandonment compared to those who do so in their place of origin (58).

However, as migrants move further away from their community during the journey, finding a “local” smuggler can get more complicated. Yet, the importance of shared nationality, ethnicity, or language does not vanish. For example, Albanian migrants have been known to purchase crossings between France and the United Kingdom from Albanian smugglers, even though the actual boat journeys were organized by an Iraqi Kurdish group (33). Anecdotal evidence suggests that Vietnamese smugglers based around Calais, in Northern France, operate in the same way (59). This type of arrangement contributes to the fragmentation of the smuggling market, resulting in the emergence of groups of different nationalities and ethnicities that specialize in different tasks. This, in turn, creates a need for increased horizontal coordination.

Financial arrangements can be designed in a way that supports transactions and overcomes some of the challenges stemming from illegality. For example, smugglers may offer to split payments into two parts, with only a deposit payable before departure (of a leg) and the rest due upon successful completion of the agreed journey (21, 49, 53). A nonrefundable deposit offers a double warranty: It signals to a migrant the smuggler’s reliability and commitment to a successful journey, but it also locks a migrant into a journey with *that* specific smuggler and not a competitor.

Splitting payments, however, raises the problem for a smuggler of how to collect the outstanding fee when the journey ends. In other words, it can (acutely) increase a smuggler’s monitoring costs. A migrant can be held hostage until full payment is received, as in the case of Chinese migrants in the United States in the 1990s (21) or the Vietnamese Witness X held in London in 2019 (discussed above). While the use of hostage-taking as a strategy to ensure commitment has a long history (60, 61), it is also very costly. Keeping people hostage might severely limit the number of migrants moved by a smuggler—and it is challenging to implement when migrants are intercepted by law enforcement. As in the case of legal online marketplaces, escrow services can offer a solution. An escrow service is an arrangement in which a third party, mutually trusted by smuggler and migrant, holds an agreed sum and only releases it when a previously agreed condition is met, i.e., when the migrant reaches the agreed destination. For instance, a migrant may deposit money with a hotel owner or shopkeeper in Türkiye, and then use a code to unlock the funds upon safely reaching Greece—indicating to the escrow agent that the smuggler has fulfilled the terms of the agreement. Informal bankers such as *hawaladars* can also serve as escrow agents in human smuggling. There are documented cases of escrow mechanisms being used across various nationalities and routes, including among migrants from the Horn of Africa, the Middle East, and Central and South Asia (26, 49, 53, 56).

Interactions between smugglers and migrants are not always straightforward and can involve significant risks, deception, and exploitation. Yet, 75% of migrants surveyed in Mexico postdeportation indicated they were satisfied with the service provided by smugglers (62, p. 153), and of the 40,215 migrants surveyed worldwide by the Mixed Migration Centre, 70% strongly agree or agree with the statement that smugglers helped them achieve their goal of migrating to another country, while only 12% disagree. Among those who have reached the end of their journey, agreement increases to 84%, and disagreement decreases to 6% (4Mi data: 11).

## 5. Enforcement, Regulations, and Policy Approaches

The rules defining legal mobility, and the related enforcement of border controls, have a direct impact on the market for human smuggling. While such rules, and the level of enforcement, might differ greatly across countries, there are some common elements that are important to note.

First, there is a clear impact of border enforcement on a) the number of migrants who seek assistance from smugglers and b) the market-clearing prices. Evidence from the US–Mexico border has shown that an increase in border enforcement leads to a parallel rise in migrants enlisting the services of a smuggler (47) and to higher prices (47, 63). Increased border enforcement has also been shown to increase risks for market participants, particularly migrants (see e.g., ref. 62 for the US–Mexico border).

Prices in the market for smuggling services appear to be sensitive to demand and supply, the perceived rate of success, and the type and quality of services provided, but also the risk faced by smugglers as a consequence of increased enforcement (49, 63, 64). Anecdotal evidence suggests that the plan announced by the UK Government to deport to Rwanda all migrants detected entering the country unlawfully on small boats prompted a market response from smugglers, who immediately cut prices considerably to entice clients, reflecting a diminished value of the service provided (65; prices went down from around £3,000 to £1,200 or even £500 after negotiations). The plan was eventually dropped and prices reportedly went up again. Driving up prices and bringing down the value of a crossing (Rwanda plan) are examples of enforcement through decreasing the market size by imposing what Reuter calls “structural consequences of product illegality” (31).

Second, there is a sense in which one could describe this market as demand-driven. Smugglers mostly respond to the demand coming from migrants rather than the other way around. Anecdotal evidence from the United States and Europe supports the idea that the smuggling market is mostly demand-driven (50, 53, 66). This is further corroborated by the responses to a multisite survey conducted by the Mixed Migration Centre (4Mi data: 11). Among those migrants interviewed in Europe at the end of a journey undertaken between 2019 and 2024, only 5% maintained that smugglers were the main influence on their decision to migrate (The top six influences were: parents (56%), friends and family in country of departure (34%), spouses (25%), friends and family in other countries (24%), social media (12%) and migrants’ children (5%). Only traditional media (1%) and returnees (1%) ranked below smugglers. Respondents could name up to three influences).

Third, policy approaches need to grapple with the fact that the demand for particular routes is subject to considerable spikes, often as a consequence of larger societal events. A striking feature of the smuggling market is that such spikes in demand *are* often satisfied by smugglers. Data from Europe are particularly telling. Successful IBCs on the Eastern Mediterranean Route into Europe (via Türkiye and Greece) increased by 1,641% between 2014 and 2015 (as a consequence, among others, of the civil war in Syria); the Central Mediterranean Route (from North Africa into Italy) increased by 1,344% between 2010–2011 and 277% between 2013–2014; small boat crossings between France and the United Kingdom went up by 516% in 2019 year-on-year, followed by +359% and +237% (Data for the Mediterranean routes are from Frontex; figures for the English Channel are published by the UK Home Office). This suggests that supply chains have a striking ability to expand quickly and adapt rapidly to surges in demand. This also suggests that capital requirements and investments in infrastructure are minimal and that, more generally, barriers to entry are low: Individuals with an entrepreneurial spirit, enough money to invest in the purchase of an inflatable boat (land crossings require even less capital investment), and a willingness to act outside the realm of the law can easily “set up shop” and start offering smuggling services. A further implication is that smugglers are likely to be easily replaceable. This makes supply-side enforcement challenging and resource-intensive.

Finally, under the UN Protocol against the Smuggling of Migrants (1), it is only the provision of smuggling services that is criminalized, with Article 5 explicitly stating that “migrants shall not become liable to criminal prosecution.” Under the Treaty’s obligations, criminalization only targets supply-side actors and not clients. In the United States, for instance, courts have recorded 4,731 cases of alien smuggling offenses in the FY 2023: The average sentence was 15 mo, and 90% of offenders were sentenced to prison (67) (Sentenced offenders were overwhelmingly men (77%), Hispanic (80%), had little or no prior criminal history (64%). Their average age was 32 y and 70% had US citizenship (67)).

The way in which States handle migrants at the border varies greatly, depending on the legal system of each country, criminal justice practices, but also the type of border (sea vs. land crossings), and other international obligations. The interplay between different pieces of domestic and international legislations can generate complex policy conundrums. For instance, migrants crossing unlawfully into a country with the assistance of a paid smuggler might have the right to log an asylum application once in the country and, if successful, obtain the lawful right to reside in that country. This generates a policy paradox: To get access to the right to asylum often a person needs first to pay the illegal services of a smuggler to physically enter the country. In the United Kingdom, 93% of people crossing the Channel on small boats claimed asylum between 2018 and March 2024, and 77% of those who had received a decision were granted refugee status or other forms of permission to stay (68). Under international law, countries have an obligation to rescue migrants at sea if in distress, and they have an obligation not to push back individuals to countries where their life or freedom would be threatened (principle of nonrefoulement). Such obligations present an inherent conflict with border control policies and enforcement, generating intense and divisive public debates, particularly in destination countries. More broadly, they highlight the conflict between fundamental humanitarian principles and the protection of individual safety and security on the one hand, and the legitimate enforcement of borders, immigration, and mobility rules on the other.

## 6. Market Developments and Innovations

Human smuggling has not changed fundamentally in nature over the years, or even decades. Nonetheless, one can observe some degree of innovation in how the market operates, primarily driven by developments in information and communication technologies.

Smugglers increasingly use mainstream social media to advertise their services and reach out to potential clients. In 2024, 8,000 accounts were removed from four major social media platforms at the request of the UK National Crime Agency alone, and a total of 16,500 over the last 3 y (69). Migrants are also increasingly using social media to identify smugglers as well as verify the information provided by smugglers. Similarly to legitimate e-commerce platforms and illegal drug cryptomarkets, online social media platforms offer a way to exchange reviews and feedback on individual smuggler, as well as to compare prices and services (see e.g., ref. 52; also ref. 4, p. 44) (Information circulating in online communities is not immune to deception and outright lies (4, p. 44)). Migrants often use a mix of online and offline sources, showing integration rather than substitution between offline and online communities (53). According to survey data from Mixed Migration Centre (11), 21% of migrants who have used a smuggler between 2019 and 2024 collected information about the journey through online communities and social media; this goes up to 29% for migrants traveling to Europe. (While technology is increasingly used, friends and family still remain an important source of information (45, 53)). Arguably, technology has made it easier for migrants to adopt a pay-as-you-go—or “à la carte”—model where they can purchase smuggling services directly from smugglers at each border without the need to engage the services of a route coordinator, potentially keeping costs down while having more control over the conditions of the journey. This, in turn, reduces the incentives for vertical integration on the smugglers’ side. In other words, technology might have led to an increase in Business-to-Customer (B2C) transactions and a decrease in Business-to-Business (B2B).

Additionally, thanks to technology, migrants have also been able to pressure smugglers to keep their word by using compromising information as a hostage—similar to what Gambetta (46) and Campana and Varese (61) have observed in other illicit contexts [see Schelling (60) on the use of hostage-taking as a general strategy to ensure commitment]. On one occasion, a Syrian migrant threatened to report a smuggler to the police using a picture secretly taken, should the smuggler refuse to organize a new crossing following a first unsuccessful attempt. The smuggler eventually agreed to the request and organized a new (successful) crossing at no additional cost (53, p. 17).

Smugglers also use technology to decrease the risk of apprehension. For instance, smugglers no longer always crew the boats crossing the Mediterranean, but rather give migrants basic training and satellite phones to track their position and call the European authorities to rescue them at sea—so the migrants can disembark on European soil (49, p. 39). Small boat crossings between France and the United Kingdom are organized in a similar fashion. Finally, technological innovations can help draw a separation between the place where the smuggling service is provided and where the financial transaction is made. In one case of smuggling across the Mediterranean, the Eritrean smuggler and his clients were in Libya while payments were made from Sweden to Israel using hawala (23). This strategy increased the safety of both migrants and smugglers by decreasing the risk of becoming victims of theft for both smugglers and migrants.

## 7. Discussion and Conclusions

Every day, across the world, thousands of individuals cross borders unlawfully with the paid assistance of professional smugglers—and thousands more wait to be smuggled or contemplate the possibility of enlisting the services of a smuggler. The market for human smuggling is both global and local. It is global, as it exists across all continents and involves individuals from many different nationalities and ethnicities. It is transnational by definition, as it involves crossing at least one international border. However, it is also strikingly local due to the significant role played by local knowledge and local communities. It is not one market, but a collection of different parallel markets. It can be broken down by route, with migrants typically moving from poorer and unsafe countries to richer and safer destinations. This movement is likely to generate revenues of billions of US dollars annually. Not all unlawful crossings involve paid smugglers, but most do—particularly when enforcement is high. Across diverse routes, smugglers adopt strikingly similar arrangements. They tend to be organized in small groups—although independent actors are also present—each characterized by a specific set of tasks and geographical specialization (often down to a single border). When present, hierarchies are rudimentary. Coordination among groups—and individuals—tends to be horizontal, with many independent actors contracted as service providers on a repeated or ad hoc basis.

Overall, such organizational arrangements give the market considerable flexibility: Capacity can be added quickly to satisfy spikes in demand. In addition, barriers to entry appear low, and the same applies to capital requirements. This makes suppressing the market an extremely challenging target for law enforcement: Smugglers are replaceable, newcomers can easily set up shop, and the segmentation of activities also means compartmentalization and, therefore, resilience. Law enforcement also requires coordination between jurisdictions. The fact that demand would still remain even if existing suppliers were taken down makes the enforcement challenge even more complex operationally but also raises moral dilemmas. How to reconcile enforcement and humanitarian assistance and protection? When increased enforcement may indirectly increase harm to migrants, how should countries that are mindful of human rights protection respond to smuggling? These are difficult questions. And they are not the only ones. Smuggled migrants are exposed to severe risks, including death, violence, and exploitation—as well as fraud. Migrants going missing while crossing deserts and shipwrecks are sadly a reality. At the same time, migrants often express high levels of satisfaction with smugglers, with many viewing them as instrumental in successfully reaching their destinations. This is the paradox of the smuggling market: Simple when it comes to organizational arrangements, skills, and resources required, but incredibly complex when it comes to harm, enforcement, and the connected moral dilemmas.

## Data Availability

There are no data underlying this work.

## Footnotes

^*^Different definitions of the term “migrant” exist; however, if we consider a migrant any person who moves away from their place of residence as per IOM lay definition, then smugglers’ clients can only be international migrants (70).

^†^Article 3, comma (a) of the UN Protocol defines “trafficking in persons” as “the recruitment, transportation, transfer, harbouring or receipt of persons, by means of the threat or use of force or other forms of coercion, of abduction, of fraud, of deception, of the abuse of power or of a position of vulnerability or of the giving or receiving of payments or benefits to achieve the consent of a person having control over another person, for the purpose of exploitation. Exploitation shall include, at a minimum, the exploitation of the prostitution of others or other forms of sexual exploitation, forced labour or services, slavery or practices similar to slavery, servitude or the removal of organs” (2).

^‡^In this paper, the terms “human smuggling” and “migrant smuggling” are used interchangeably.

^§^Data for the US Southern Border are from the CBP Public Data Portal, cpb.gov.

^¶^This figure only includes “apprehensions” under Title 8 of U.S.C. 1226, this is the standard US immigration law governing detention, processing, and removal of migrants who enter or remain in the United States unlawfully. During the Covid pandemic, a public health measure (Title 42 U.S.C 265) was exceptionally invoked to immediately expel migrants at the border without processing them under immigration laws. A total of 2.9 million Title 42 expulsions were carried out from March 2020 to May 2023; it is possible that a part of those individuals used a smuggler to cross the border.

^#^Data from Frontex, the European Coast Guard and Border Agency, publicly available at https://www.frontex.europa.eu.

^||^Fees were estimated to be US$5,000 on average from Mexican migrants and US$7,000 for non-Mexicans ((4), p. 99). More recently, Chief Patrol Agent Aaron Heitke, San Diego Sector mentioned US$8,000 as the average fee in a testimony before the House Committee on Homeland Security. Transcript available at https://homeland.house.gov/2023/12/14/now-nobody-crosses-without-paying-senior-border-patrol-agents-describe-unprecedented-cartel-control-at-southwest-border.

^**^According to the Director of the French Office for the Fight against Illicit Trafficking of Migrants, interviewed by Infomigrant (https://www.infomigrants.net/en/post/50834/inside-frances-battle-against-migrant-smugglers-a-closer-look). This is based on a price of between €2,500–€3,000 per person.

^††^See e.g., MMP 174 Database (the Mesoamerican Migration Project, formerly known as the Mexican Migration Project): https://mmp.research.brown.edu/mmp.

^‡‡^4Mi surveys migrants who have engaged in irregular migration during at least parts of their journeys. Data presented in this work are calculated based on migrants who have made their journey between 2019 and 2024.

^§§^This modus operandi is confirmed by another UK-based Vietnamese smuggler who, under the condition of anonymity, has explained to the BBC that his work consisted of forging the documentation to get legitimate work visas to Eastern European countries. Migrants would then continue on to Western Europe and the United Kingdom, although the smuggler interviewed did not provide any evidence of his involvement in the subsequent stages of the journey (71).

^¶¶^https://www.theguardian.com/uk-news/2021/jan/22/essex-lorry-deaths-two-ringleaders-jailed-total.

^##^https://www.infomigrants.net/fr/post/52632/quatre-ans-plus-tard-le-proces-du-camion-de-lhorreur-souvre-a-paris.
